# Mutational analysis of the potential catalytic residues of the VV G1L metalloproteinase

**DOI:** 10.1186/1743-422X-3-7

**Published:** 2006-02-27

**Authors:** Kady M Honeychurch, Chelsea M Byrd, Dennis E Hruby

**Affiliations:** 1 Department of Microbiology, Oregon State University, Corvallis, Oregon 97331, USA; 2 SIGA Technologies, Inc, Corvallis, Oregon 97333, USA

## Abstract

The vaccinia virus G1L open-reading frame is predicted to be a metalloproteinase based upon the presence of a conserved zinc-binding motif. Western blot analysis demonstrates G1L undergoes proteolytic processing during the course of infection, although the significance of this event is unknown. In order to determine which amino acid residues are important for G1L activity, a plasmid-borne library of G1L constructs containing mutations in and about the active site was created. Transient expression analysis coupled with a *trans *complementation assay of a conditionally-lethal mutant virus suggest that, of the mutants, only glutamic acid 120 is non-essential for G1L processing to occur.

## Findings

Vaccinia virus (VV) is among the largest of the DNA viruses and represents the prototypic member of the Orthopoxvirus genus. It is an enveloped virus and possesses a linear double-stranded genome containing greater than 200 mostly non-overlapping open reading frames. Throughout its life cycle, VV replicates exclusively in the cytoplasm of infected cells, although the presence of a nucleus is required in order for the virus to mature properly [1,2]. During replication, VV undergoes three distinct stages of gene expression, the products of which are referred to as early, intermediate and late proteins. In general, early proteins are components of the replication machinery, intermediate proteins assist in the transcription of late proteins and late proteins consist of the virion structural elements, the trafficking and assembly of which are regulated by modifications such as acylation, myristoylation and palmitoylation [3-8] as well as by host cell and virally encoded proteases.

**Figure 1:**
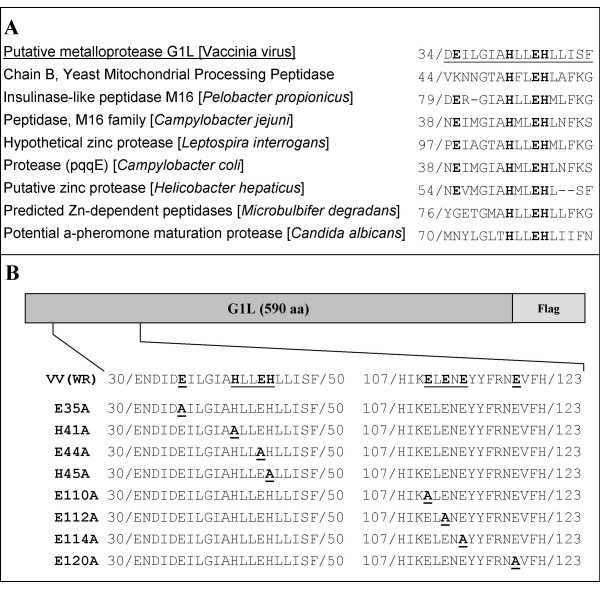
(A) Alignment of the VV G1L putative catalytic domain with the catalytic domain found throughout MMPs. (B) G1L mutant library. Schematic of alanine substitutions within the G1L ORF. Each construct includes a C-terminal Flag epitope for detection by Western blot analysis.

**Figure 2:**
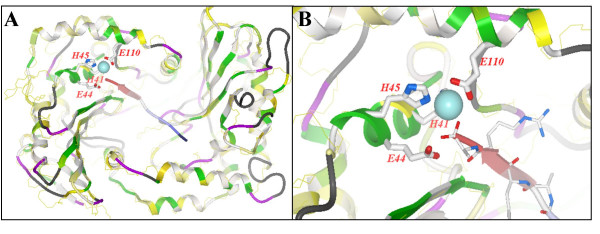
Homology model of G1L using the yeast MPP as a template. G1L is depicted as a ribbon colored by alignment: identical residues, green; similar residues, yellow; non-conserved residues, white; insertions, magenta; deletions, black. The MPP template is shown as a thin yellow line. Active site residues are represented as thick sticks and the Zn^2+ ^ion as a cyan sphere. A substrate peptide is shown as blue and red ribbon and thin sticks. (A) G1L in its entirety. (B) Close-up view of the putative active site residues.

The complete DNA sequence of VV revealed the presence of two potential proteinases, the products of the I7L and G1L open reading frames [9]. The first, I7L, was originally identified by limited sequence similarity to a ubiquitin-like proteinase in yeast [10]. I7L is now recognized as the VV core protein proteinase and is at least one of the entities responsible for initiating the morphogenic transformation of immature virus (IV) particles into intracellular mature virus (IMV) [11-13] and is currently a target of rational antiviral drug design [14]. The second apparent proteinase is G1L. G1L was initially thought to be the proteinase responsible for the late-stage proteolytic morphogenesis of at least one of the viral core proteins based upon results obtained from a transcriptionally controlled *trans*-processing assay [15] G1L contains a canonical HXXEH zinc-binding motif [16], which is a direct inversion of the established HEXXH motif found in a wide array of matrix metalloendopeptidases (MMPs), including thermolysin [17], aminopeptidase N [18] and collagenase [19]. The particular sequence contained within G1L is characteristic of the M16 (pitrilysin) family of MMPs as well as a variety of proteins found in bacteria and yeast (Fig. 1A). Most known MMPs include a signal sequence to allow for secretion, an inhibitory pro-sequence to regulate activity and a catalytic domain containing a catalytically active Zn^2+ ^ion [20]. Amino acid sequence analysis demonstrated very little similarity between G1L and other MMPs, aside from the presence of a potential zinc-binding motif. However, computational modeling has revealed that structurally, G1L appears to contain a significant likeness to the β-subunit of the yeast mitochondrial processing peptidase (MPP), which is the closest structural homolog with an available X-ray structure [PDB:1hr9.b] (Fig. 2) [21,22]. The β-subunit of the yeast enzyme is characterized by the presence of an inverted zinc-binding motif, HXXEHX_n_E, where two histidine residues and a distal glutamic acid (E) residue coordinate a zinc cation [16,23,24]. The E residue within the HXXEH motif is involved in peptide bond hydrolysis through the activation of water [23,25]. The essential downstream E residue for M16 MMPs is found within a region containing several completely conserved E residues [23]. Although G1L lacks an exact match to this region, it does contain a region 65 residues downstream of the HXXEH sequence consisting of ELENEX_5_E (residues 110 through 120) that is very highly conserved among poxviruses.

While it is tempting to predict that VV G1L behaves in a manner similar to yeast MPP, the fact remains that very little is actually known about G1L activity. Through the development of a conditional-lethal recombinant vaccinia virus, G1L was identified as an essential component of the VV replication cycle [26,27]. Conditional-mutants grown under non-permissive conditions arrested their replication subsequent to core protein cleavage but prior to complete core condensation suggesting the major viral proteins are expressed and processed independently of G1L but that G1L plays a crucial role in the conversion of vaccinia virus from immature virions into infectious IMV particles [26-28]. Western blot analysis suggests G1L exists initially as a 68 kDa entity, which may be cleaved into 46 kDa and 22 kDa products [26]; however, the significance of this cleavage remains unclear. The presence of a bound zinc ion has yet to be experimentally confirmed, although efforts to obtain G1L in sufficient quantities and of sufficient purity to allow for such analyses are currently underway.

In the present study, an analysis of the putative active site of G1L was carried out in an attempt to understand which amino acid residues are important for G1L processing as well as which of the four downstream E residues present within the highly conserved ELENEX_5_E sequence is likely to participate in the coordination of a zinc ion if such an interaction does in fact occur. Through the use of a library of transiently expressed G1L mutants containing C-terminal flag epitopes coupled with rescue analysis of a tetracycline-dependent conditional-lethal mutant, the results obtained suggest that only E120 can withstand mutation and still produce a phenotype similar to what is observed for wild type G1L.

The putative active site of VV G1L is thought to consist of histidine (H) residues 41 and 45 and a downstream E, all of which are thought to contribute to zinc-binding, and E residue 44, which is predicted to participate in the hydrolysis of the substrate peptide bond. In this study, each of these residues was systematically mutated to alanine (A) (Fig 1B). There are four candidate downstream E residues, including E110, E112, E114 and E120. Computational modeling utilizing the β subunit of the yeast MPP suggests E110 is the downstream E residue involved in zinc coordination (Fig. 2). Mutation of E112 or E114 was shown to abrogate the processing of p25K in a *trans*-processing assay [15], although whether or not this result was an artifact of the assay remains to be determined. The fact that both E112 and E114 are very highly conserved throughout the Orthopoxvirus genus as well as poxviruses in general implies that at least one of them may be involved in the activity of G1L. Glutamic acid residue 120 was selected for mutation since its location 74 residues downstream from the HXXEH motif follows the same pattern observed for members of the zinc-dependent M16 family of metalloproteinases [29,30] as well as a variety of other metalloenzymes (Fig 1A). These enzymes are characterized by an HXXEHX_74–76_E active site motif, a motif that could apply to G1L if the zinc-binding downstream residue is E120. E35, a highly conserved residue not predicted to participate in the catalytic activity of G1L, was also mutated in order to gain an understanding of the roles played by other charged residues that surround the active site. A study conducted by Kitada et al [23] demonstrated that a conserved E residue just upstream of the active site motif functioned as a necessary acidic residue since mutational analysis demonstrated a loss of enzyme activity upon the replacement of E47 with A but a partial restoration of activity when an aspartic acid (D) was substituted for E47. Each construct in this study was engineered with a flag epitope (DYKDDDDK) on the C-terminus for detection in immunoblot analysis (Fig. 1B) and was placed under the control of either the synthetic early-late promoter [31], as in the case of constructs used in transient expression assays, or the native G1L promoter, which is located in the region 229 basepairs upstream of the G1L initiating codon, for constructs employed in rescue assays.

**Figure 3:**
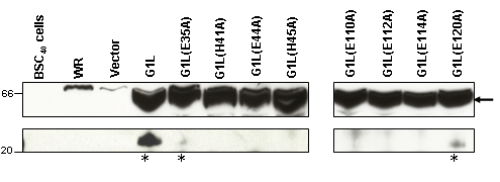
G1L undergoes proteolytic processing. BSC_40 _cells were transfected with 1.5 μg of plasmid DNA containing either wild type G1L or one of the eight single-site mutants. Four hours later, the transfection solution was removed and cells were infected with VV strain WR at an MOI of two. Twenty-four hours later, cells were harvested and extracts were subjected to Western blot analysis with anti-Flag antisera. The top panel demonstrates full length G1L, indicated by the arrow, and the lower panel shows the resulting cleavage products indicated by *.

Previous studies have demonstrated that during the VV replication cycle G1L undergoes an internal cleavage event [26]. At this point it remains unclear as to whether G1L participates in autoproteolysis or exists as a substrate of another viral or cellular proteinase. In an effort to get one step closer to answering this question, the fate of the G1L mutant plasmid library was analyzed in the context of a VV infection. BSC_40 _cells [1] were transfected with 1.5 μg of plasmid DNA containing either wild type G1L or one of the eight single-site mutants by way of a liposome-dependent transfection procedure. Four hours later, the transfection solution was removed and cells were infected with VV strain Western Reserve (WR) at a multiplicity of infection (MOI) of two. Twenty-four hours later, cells were harvested and extracts were subjected to immunoblot analysis using anti-flag antisera. Results indicate that in each case full length G1L is expressed at approximately 66 kDa as indicated by the arrow (Fig. 3, upper panel). However, a cleaved product is only observed for wild type G1L and G1L containing a mutation at E120. The mutant construct E35A also appears to undergo some degree of cleavage albeit to a much lesser extent (Fig. 3, lower panel). Taken together, these results suggest that alterations in the amino acid sequence of the putative active site of G1L as well as three of the four potential downstream zinc-binding residues renders the protein either unable to perform autocatalysis or unrecognizable as a substrate for another proteinase. Additionally, these results imply that E120 would not be the downstream residue involved in the coordination of a zinc ion.

**Figure 4:**
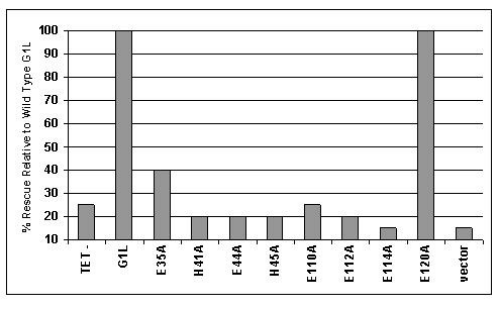
Mutational analysis of the putative catalytic and zinc-binding residues of G1L utilizing a *trans *complementation assay. TREx 293 cells were transfected with 1 μg plasmid DNA bearing either wild type G1L or one of the single-site mutants. Four hours later the transfection solution was removed and replaced with infection media containing vvtetO:G1L at an MOI of 0.1 Cells were harvested at twenty-four hours post infection, subjected to a series of rapid freeze/thaws and titered via plaque assay on BSC_40 _cells. Bars represent the percent rescue of each construct relative to what was achieved by transfection with the wild type G1L construct (G1L). Each transfection was carried out in the absence of TET (TET-).

To determine the role of these conserved residues in G1L activity, the mutant G1L constructs were evaluated for their ability to rescue viral replication in a *trans *complementation assay utilizing a tetracycline (TET)-dependent recombinant VV, vvtetO:G1L. The construction of vvtetO:G1L is described in detail in 27. Briefly, the TET operator was inserted directly upstream of the G1L open reading frame. When used in conjunction with a commercially available cell line expressing the TET repressor, expression can be controlled by either the presence or absence of TET within the culture media. In our assay, TREx 293 cells (Invitrogen, Carlsbad, CA) were transfected with 1 μg plasmid DNA bearing either wild type G1L or one of the single-site mutants. Four hours later the transfection solution was removed and replaced with infection media containing vvtetO:G1L at an MOI of 0.1 Cells were harvested at twenty-four hours post infection, subjected to a series of rapid freeze/thaws to release intracellular virus particles and titered by plaque assay on BSC_40 _cells. Figure 4 shows the average percent rescue obtained for each of the mutant constructs relative to wild type G1L and is the culmination of two independent experiments. With the exception of wild type G1L, the E120A mutant and, to a lesser extent the E35A mutant, none of the other mutants were capable of complimenting the conditional mutant virus grown in the absence of TET. These results are in accordance with what was observed in the transient processing assay and suggest that cleavage of G1L is a necessary event in order for VV to propagate efficiently. The fact that the E35A construct conveys only partial rescue correlates with the reduction in proteolysis observed by immunoblot.

With the recent identification of I7L as the VV core protein proteinase [11-13], the function of G1L within the viral replication cycle remains an enigma. Initially, the core proteinase was thought to be G1L due to discovery of an HXXEH inverted zinc-binding motif common to members of the M16 family of metalloproteinases (Fig. 1A). This motif is 100% conserved among poxviruses implying it is the enzyme active site. Additional sequence analysis and structural modeling further support the hypothesis that G1L may be the first example of a virally encoded metalloenzyme, although, G1L has not yet been shown to coordinate a zinc ion. To date, G1L expression is known to be an essential part of a VV infection. It has also been shown to undergo proteolytic processing, which appears to be essential for activity.

In this manuscript we report on the analysis of eight highly conserved residues within VV G1L including the three conserved residues located within the HXXEH catalytic motif as well as four downstream E residues, one of which is believed to be the essential downstream residue involved in the coordination of a zinc ion (Fig. 2). A library of C-terminally flag-tagged G1L constructs containing single point mutations to each residue was generated (Fig. 1B). Transient expression assays allowed us to monitor the processing of each construct through the detection of a C-terminal cleavage product. Cleavage was observed for wild type G1L as well as the construct containing a mutation to E120 (Fig. 3), however G1L appeared unable to tolerate mutations in E110, E112 or E114. Further, mutations in H41, E44 or H45 also inhibited G1L processing. This may be indicative of either the loss of a zinc ion, which would render a metalloproteinase inactive or the destabilization of protein folding resulting in the inability of G1L to be recognized as a substrate. Interestingly, mutation of E35, a highly conserved residue not predicted to participate in zinc-binding or substrate association, appears to affect G1L activity as well, although these effects were less dramatic than what was observed for the other mutant constructs as evidenced by the presence of a very faint C-terminal product. The lack of a robust cleavage reaction suggests the presence of an alanine at this position may destabilize the protein structure enough to make processing inefficient. Furthermore, rescue of a conditionally lethal mutant virus grown under non-permissive conditions was only observed with the E120A mutant (Fig. 4), which produced a phenotype nearly identical to the phenotype observed for wild type G1L. The E35A mutant also demonstrated the ability to rescue; however, rescue was markedly diminished relative to wild type G1L. In no other case did rescue reach above 25% of wild type.

These results suggest that G1L may fit into the paradigm of what is observed for other metalloproteinases and proteolytic enzymes in general in that G1L may initially be translated as an inactive zymogen, which is then activated upon proteolysis. In the absence of G1L expression, arrest in replication occurs subsequent to the activities of I7L, but prior to complete core condensation [27] suggesting the involvement of G1L in a proteolytic cascade. Of course these data do not rule out the possibility that the processing observed is simply an artifact of over-expression similar to what is observed for membrane-type-1 MMP, which may undergo autoproteolysis to an inactive form as a means of negative regulation in response to conditions of over-expression [32]. This scenario is unlikely, however, since the C-terminal region of G1L was observed via silver stain in the context of a conditional mutant VV infection [26].

The role of both the full-length and cleaved products of VV G1L continue to be investigated as does the ability of G1L to coordinate a zinc ion. If G1L is recognized as a bona-fide metalloproteinase, it will be of interest to determine if it acts alone or has a requirement for an additional subunit, much like what is observed for other MPPs. Further, once an appropriate expression system is established, analysis via mass spectroscopy may be used to determine exactly where G1L cleavage occurs, which may in turn aid in the identification of potential G1L substrates.

## Competing interests

The author(s) declare that there are no competing interests.

## Authors' contributions

KMH conducted the experiments and wrote the manuscript. CMB constructed the conditional lethal virus, assisted with the creation of the figures and edited the manuscript. DEH conceived the study, coordinated the research efforts and edited the manuscript. All authors read and approved of the final manuscript.
